# Bioactive Properties of Algerian Bee Pollen: Influence of Botanical Origin on Polyphenol Content and Antioxidant Capacity

**DOI:** 10.3390/foods15020202

**Published:** 2026-01-07

**Authors:** Yasmine Saker, Olga Escuredo, María Carmen Seijo, Sonia Harbane, Akli Ouelhadj, María Shantal Rodríguez-Flores

**Affiliations:** 1 GISA—Grupo de Investigación en Sistemas Agroambientales, Departamento de Biología Vegetal y Ciencias del Suelo, Facultad de Ciencias, Universidade de Vigo, 32004 Ourense, Spain; yasmine.saker@uvigo.gal (Y.S.); mcoello@uvigo.gal (M.C.S.); sonia.harbane@uvigo.gal (S.H.); mariasharodriguez@uvigo.gal (M.S.R.-F.); 2 Instituto de Agroecoloxía e Alimentación (IAA), Universidade de Vigo, Campus Auga, 32004 Ourense, Spain; 3 Ecology, Biotechnology and Health Laboratory, Faculty of Biological Sciences and Agronomic Sciences, University of Mouloud Mammeri, Tizi-Ouzou 15000, Algeria; akli.ouelhadj@ummto.dz

**Keywords:** bee pollen, palynology, phenols, flavonoids, minerals, radical scavenging activity

## Abstract

The composition and biological activity of bee pollen are strongly influenced by its botanical origin, which is determined by the regional flora and environmental conditions. In Algeria, despite the growing consumption and traditional use of bee products, especially in the Mediterranean region known for its rich floral biodiversity, scientific studies on bee pollen remain scarce. This study aims to characterize bee pollen from the Mediterranean region of Algeria by identifying its botanical origin and evaluating its total phenolic content, total flavonoid content, antioxidant activity, and minerals. A total of 27 bee pollen samples were collected and subjected to palynological analysis to determine their floral sources. Total phenolic content and total flavonoid content were determined by standard colorimetric assays, and antioxidant activity was assessed using RSA with a DPPH assay, ABTS^+^•, and FRAP methods. The results revealed significant variability in the phenolic composition and antioxidant capacity of the samples, depending on their botanical origin. Pollen types such as *Brassica napus* type, *Acacia*, *Myrtus communis*, and *Sinapis alba* showed notably higher phenols, flavonoids, and antioxidant activity, whereas *Hedysarum* and *Daucus carota* pollen exhibited the lowest values. The mineral profile, including macro- and microelements (such as K, Ca, Mg, P, Fe, Cu, Mn, and Na), was determined to explore the nutritional value and potential correlations with biochemical parameters. These findings highlight the influence of floral biodiversity on the bioactive potential of bee pollen and underscore the value of Algerian bee pollen as a promising source of natural antioxidants.

## 1. Introduction

Pollen is the main food source for the larvae of many insects. Honey bees gather pollen grains from plants and mix them with nectar and salivary gland secretions to form pollen pellets. These pollen pellets are transported by bees’ legs and collected by beekeepers using pollen traps placed at the entrance of beehives [1,2]. Bee pollen is a floral mixture whose morphology, size, shape, and color diverge according to the plant species visited by honeybees [3]. Pollen pellets exhibit differences in symmetry, exine structure, and sculpture. For example, the pollen types belonging to the Asteraceae family are spinulose, those belonging to the Malvaceae family are echinate, pollen grains belonging to the Myrtaceae family are colporate and prolate, those belonging to the Euphorbiaceae family are inaperturate and reticulate, and species belonging to the Fabaceae family show variability in their pollen type [4]. Different colors are attributed to the pollen loads according to the plant species visited by honeybees, such as white, yellow, orange, red, purple, brown, and black [5].

Bee pollen is considered a perfect nutrient due to its richness in various natural substances [6,7,8]. It is a nutrient-rich product with a complex composition including carbohydrates, proteins, lipids, vitamins, phenolic compounds, and minerals that are essential for honey bees and humans alike [5,9,10]. Bee pollen’s main chemical components are carbohydrates (13–55%), proteins (10–40%), lipids (1–10%), and crude fibers (0.3–20%) [11]. Other minor components, such as minerals, vitamins (A, D, E, and C), bioflavonoids, amino acids, phenols, flavonoids, carotenoids, and tannins, are also present in this matrix [12]. These compounds have been reported to exhibit various biological activities in bee pollen [13,14]. However, the composition of bee pollen varies greatly depending on plant species, geographical origin, climatic conditions, soil types, and beekeeping practices [15,16].

For centuries, bee pollen has attracted increasing scientific interest due to its potential health-promoting properties. It has been used in different countries as a traditional medicine to treat various diseases, including cardiovascular disease, gastric disease, tumors, diabetes, and neurodegenerative disease [17,18]. Recently, the different biological activities of bee pollen have been studied, including anti-inflammatory, antibacterial, antitumor, hypolipidaemic, hepatoprotective, antidiabetic, and anti-mutagenic activities [3,6]. In addition to its various biological activities, bee pollen, like other bee products, is a rich source of antioxidants. These antioxidants are protective agents that reduce oxidative damage to important biomolecules, including lipoproteins and DNA (deoxyribonucleic acid), from ROS [19]. The presence of antioxidants in bee pollen reduces the effects of free radicals in cells and decreases the risk of degenerative diseases by reducing oxidative stress and the oxidation of food [7,16]. The antioxidant properties of bee pollen have been attributed to the activity of antioxidant enzymes (superoxide dismutase, catalase, and peroxidase), as well as the presence of low-molecular antioxidants such as carotenoids, tocopherols, ascorbic acid, and phenolic substances [20]. Various methods have been employed to assess the antioxidant capacity of bee pollen, including enzymatic and non-enzymatic approaches. Non-enzymatic methods, such as DPPH (2,2-diphenyl-picrylhydrazyl hydrate), ABTS (ácido 2,2′-azino-bis(3-etilbenzotiazolina-6-sulfónico)), and FRAP (Ferric Reducing Antioxidant Power assay), are the most commonly used for measuring free scavenging activity [19]. In addition to the metabolites that interfere with free radical scavenging activities, the abundance of phenolic acids and flavonoids has been shown to be responsible for most biological properties [7,12]. However, the concentration of these compounds in bee pollen varies depending on the botanical species [21,22].

Bee pollen includes a wide range of minerals [9,10]. The mineral fraction, though quantitatively smaller than other constituents, plays a critical role in determining the nutritional value and physiological functionality of bee pollen. Potassium (K) and phosphorus (P) are generally the predominant elements, followed by magnesium (Mg) and calcium (Ca), while trace elements such as iron (Fe), zinc (Zn), manganese (Mn), and copper (Cu) are found in lower but biologically relevant amounts [10,23,24]. These minerals are essential for cellular homeostasis, enzymatic activity, and oxidative balance, contributing to the antioxidant capacity and metabolic regulation in both honey bees and consumers [25,26]. However, their concentrations are highly influenced by botanical origin, geographical conditions, soil composition, and harvesting season, as plants accumulate mineral salts differently depending on their physiology and environment [9,10,27]. Therefore, understanding the mineral profile of bee pollen is crucial not only for assessing its nutritional quality but also for identifying its botanical and geographical origins and potential use as a biomarker of environmental conditions [10,26].

In recent years, the consumption of bee products, especially bee pollen, has increased all over the world due to their nutritional and health benefits, which has increased their economic, social, and environmental value [28,29]. Since ancient times, bee products and beekeeping have played an important role in Algeria because they have been used in traditional medicine. Algeria is the largest country in Africa. It covers an area of 2.4 million km^2^, most of which consists of the Sahara Desert and steppe. The Mediterranean region of Algeria represents only 4% of the total land area [30,31], and is characterized by significant plant biodiversity is characterized. The most frequently found species in this diversity are Myrtaceae (*Myrtus communis*), Apiaceae *(Daucus, Coriandrum, Foeniculum, Pimpinella*), Fabaceae (*Hedysarum*, *Trifolium*), Asteraceae (*Galictites*, *Anthemis*), and Brassicaceae (*Brassica*, *Sinapis*) [32]. In the last decade, these products have become indispensable and have gained importance in terms of human consumption in Algeria [33]. It has been documented that this activity is particularly relevant in the Mediterranean region due to the climate and high biodiversity [30]. However, despite the significant floral diversity and the daily use of bee products in Algeria, few studies have been conducted on the composition of bee pollen. Thus, this study aims to characterize bee pollen collected from the Mediterranean region of Algeria by investigating its botanical origin, total phenolic and flavonoid content, antioxidant properties, and mineral content. Furthermore, the study evaluated the influence of floral biodiversity on these parameters, providing insight into the relationship between the botanical source and the bioactive potential of bee pollen.

## 2. Materials and Methods

### 2.1. Reagents and Chemicals

All reagents and chemicals used in this study were of analytical grade or higher purity. Absolute ethanol (≥99.5%, ACS reagent grade, CAS No. 64-17-5) used for the preparation of hydroalcoholic extracts was obtained from Sigma-Aldrich (St. Louis, MO, USA). Distilled and ultrapure deionized water was used throughout all analyses. Folin–Ciocalteu phenol reagent, gallic acid, quercetin, aluminum chloride (AlCl_3_), sodium carbonate (Na_2_CO_3_), sodium hydroxide (NaOH), ferric chloride hexahydrate (FeCl_3_·6H_2_O), 2,4,6-tripyridyl-s-triazine (TPTZ), sodium acetate trihydrate, and Trolox (6-hydroxy-2,5,7,8-tetramethylchroman-2-carboxylic acid) were purchased from Sigma-Aldrich (St. Louis, MO, USA) and Thermo Scientific (Waltham, MA, USA). Bovine serum albumin (BSA) standard solution and Coomassie Brilliant Blue G-250 reagent used for protein quantification by the Bradford method were obtained from Sigma-Aldrich (St. Louis, MO, USA). 2,2′-Azino-bis (3-ethylbenzothiazoline-6-sulfonic acid) diammonium salt (ABTS, ≥98%) was supplied by Thermo Scientific (Waltham, MA, USA). Potassium persulfate (K_2_S_2_O_8_, analytical grade), used for the generation of the ABTS^+^• radical, was obtained from Carlo Erba Reagents (Val de Reuil, France). Concentrated nitric acid (HNO_3_, 65%) and hydrogen peroxide (H_2_O_2_, 30%) used for microwave-assisted mineral digestion were of suprapure grade and obtained from commercial suppliers. Certified multi-element standard solutions (1000 mg/L) used for mineral calibration were prepared in nitric acid medium. Buffer solutions (pH 4.0 and 7.0) used for pH-meter calibration were supplied by Hach (Düsseldorf, Germany).

### 2.2. Bee Pollen Samples

**Figure 1:**
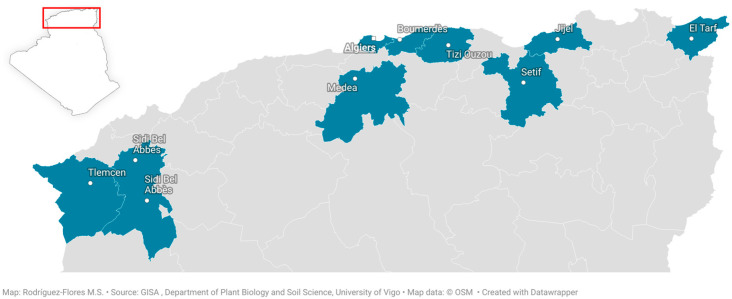
Geographical area of bee pollen samples of Mediterranean region of Algeria. Created with Datawrapper.

This present work was carried out on 27 bee pollen samples from the Mediterranean region of Algeria. The samples were collected during the period of February to October 2022, and were stored at −19 °C until analysis. The geographical origin of the samples is shown in Figure 1.

### 2.3. Palynological Analysis

Palynological analyses were carried out to determine the botanical origin of the collected samples. After homogenization, each sample (1 g) was weighed, and then a colorimetric separation was performed to obtain a subsample of a different weight [12]. This sub-sample was then fully dissolved in 5 mL of distilled water. The solution was then centrifuged for 10 min at 4500 rpm. After centrifugation, 10 µL of the sediment was taken to prepare the slides. Identification of the pollen types found in each sample was achieved through optical observation using a Nikon Optiphot II microscope (Nikon Instruments Europe B.V., Amsterdam, The Netherlands). Pollen types were determined at the family and genus levels. The pollen spectrum of each sample was determined by considering the relative weight of each color-based subsample and its botanical composition. The results were expressed as %.

### 2.4. Preparation of Bee Pollen Extracts for Analysis

Bee pollen extracts were prepared following the method described by Gabriele et al. [34], with slight modifications. For each sample, 0.5 g of bee pollen was weighed accurately and dissolved in 50 mL of 80% ethanol (prepared by mixing 100 mL of distilled water with 400 mL of ethanol) to achieve a final concentration of 0.01 g/mL. Extraction was performed in duplicate for each sample.

The mixtures were placed in 50 mL centrifuge tubes, kept in the dark, and gently agitated using a horizontal orbital shaker (SBS model MOC-30, Steinberg Systems, Berlin, Germany) at 70–90 rpm for 5 h. After agitation, the samples were macerated for 24 h in the dark to allow for the complete extraction of phenolic compounds. After maceration, the extracts were centrifuged at 4500 rpm for 10 min using a Sigma Laborzentrifuge 3–10 centrifuge (Sigma Laborzentrifugen GmbH, Osterode am Harz, Germany). The resulting clear liquid was carefully transferred to amber glass containers to prevent light exposure and stored at 4 °C until further analysis.

### 2.5. Assessment of Physicochemical Parameters: Water Content, Protein Content, and pH

The water content of bee pollen was determined gravimetrically based on the loss of mass by desiccation until constant weight was reached. Approximately 2 g of homogenized pollen were accurately weighed in pre-dried containers, previously tared. The samples were dried in a forced-air oven at 100 °C for 12 h, cooled in a desiccator for 1 h, and then weighed. The drying and weighing process was repeated until two consecutive measurements were constant. The water content was calculated as a percentage according to the following equation:

$$\mathrm{Water}\;(\%)\;=\;\frac{m-mf}{m-m0}\;\times 100$$

where *m* is the mass of the container and sample before drying, *mf* is the mass after drying (constant weight), and *m*0 is the mass of the empty container.

The total protein content of the bee pollen samples was determined by the Bradford method [35] using a commercial Coomassie Brilliant Blue G-250 reagent. Pollen extracts were prepared by dissolving 0.1 mg of the sample in 25 mL of NaOH 0.1 M (4.0 mg/mL), followed by centrifugation and recovery of the supernatant. The absorbance of the reaction mixture was measured at 595 nm using bovine serum albumin (BSA) as a standard. A calibration curve (y = 0.6884x + 0.0268; R^2^ = 0.987) was used to calculate protein concentrations, which were expressed as g protein/100 g dry weight (DW).

The pH of bee pollen samples was determined following the procedure described by Feás et al. [29], with slight modifications. A suspension was prepared by dispersing 5 g of bee pollen in 20 mL of distilled water and homogenizing at room temperature. The pH was measured using a digital pH/mV/conductivity/temperature meter (model HI 9811, Hanna Instruments, Smithfield, RI, USA), previously calibrated with standard buffer solutions at pH 4.0 and 7.0.

### 2.6. Assessment of Total Phenolic and Flavonoid Content

The total phenolic content (TPC) was measured using the Folin–Ciocalteu method adapted to bee pollen [36]. A 1 mL aliquot of the ethanolic extract was mixed with 10 mL of distilled water, 1 mL of Folin–Ciocalteu reagent, and 4 mL of 7% sodium carbonate (Na_2_CO_3_) solution. The mixture was brought to a final volume of 25 mL with distilled water and incubated for 1 h at room temperature in the dark. Absorbance was measured at 765 nm using a UV–Vis spectrophotometer (Jenway 6305, Fisher Scientific, Loughborough, UK). Gallic acid (GA) was used as a standard for calibration. The standard curve was constructed using concentrations ranging from 0.01 to 0.50 mg/mL, following the equation Abs = 84.641C + 0.0152 (R^2^ = 0.998). Results were expressed as mg gallic acid equivalents per gram of bee pollen extract (mg GAE/100 g).

Total flavonoid content (TFC) was determined according to the method of Arvouet-Grand et al. [37]. In total, 2 milliliters of bee pollen extract (0.01 g/mL) were mixed with 0.5 mL of 5% aluminum chloride (AlCl_3_) solution in methanol and diluted to 25 mL with distilled water. The mixture was incubated for 30 min in darkness, and absorbance was measured at 425 nm using a UV–Vis spectrophotometer (Jenway 6305, Fisher Scientific, Loughborough, UK). Quercetin was used as the reference standard to construct the calibration curve (0.002–0.010 mg/mL), following the equation Abs = 78.232C + 0.00461 (R^2^ = 0.998). Results were expressed as mg quercetin equivalents per gram of bee pollen extract (mg QE/100 g).

### 2.7. Assessment of Antioxidant Activity

The antioxidant activity of bee pollen samples was evaluated using three analytical methods. The 2,2-diphenyl-picrylhydrazyl hydrate (DPPH) scavenging method was realized using the method developed by Sánchez-Moreno [38] and Tabart et al. [39]. A total of 0.3 mL of bee pollen extract (0.01 g/mL) was mixed with 2.7 mL of (6 × 10^−5^) a DPPH (2,2-diphenyl-1-picrylhydrazyl) solution. The bee pollen sample solution and the blank DPPH solution were incubated in the dark at room temperature for 30 min, and the absorbance was measured at 517 nm using a UV-Vis spectrophotometer. The discoloration of DPPH in each sample tested was calculated by the percentage of RSA (radical scavenging activity) using the following formula:

$$\mathrm{RSA}\;\%\;=\;\frac{\left(A_{0}-A_{s}\right)}{A_{0}}\times 100$$

where

$A_{0}$ = absorbance of the control (DPPH solution without sample);

$A_{s}$ = absorbance of the sample (DPPH solution with bee pollen extract);

A higher RSA % value indicates greater free radical scavenging activity, reflecting stronger antioxidant capacity of the sample.

The ABTS^+^• ([2,2′-azino-bis (3-ethylbenzothiazoline-6-sulfonic acid)] radical scavenging assay was determined by using the method of Re et al. [40]. The ABTS radical cation (ABTS^+^•) solution was prepared by reacting ABTS 7 mM dissolved in water with 2.45 mM potassium persulfate. The ABTS stock solution was left in the dark at room temperature for 12–16 h until stabilization. The ABTS^+^• solution was diluted with ethanol to obtain an absorbance of 0.70 ± 0.02 at 734 nm. 980 µL of the ABTS solution was mixed with 20 µL of the ethanolic extract of the bee pollen samples. The absorbance was measured at 734 nm. The percentage of radical scavenging activity (RSA) of ABTS^+^• was calculated using the following formula:

$$\mathrm{ABTS}\;\mathrm{Inhibition}\;(\%)\;=\;\frac{\left(A_{0}-A_{s}\right)}{A_{0}}\times 100$$

where

$A_{0}$ = absorbance of the ABTS^+^• solution (control);

$A_{s}$ = absorbance after reaction with the bee pollen extract;

This method provides a broad evaluation of both hydrophilic and lipophilic antioxidant compounds.

The Ferric Reducing Antioxidant Power Assay (FRAP) was measured following the method of Benzie and Strain [41] adapted to bee pollen. The FRAP solution contains 10 mL of acetate sodium trihydrate (300 mM), 1 mL of TPTZ (10 mM) solution, and 1 mL of ferric chloride (20 mM) solution. In total, 100 µL of bee pollen extract solution was mixed with 4 mL of FRAP reagent, and the solution was incubated at 37 °C in a water bath for 4 min. The absorbance was measured at 593 nm using a UV–Vis spectrophotometer (Jenway 6305, Fisher Scientific, Loughborough, UK). The FRAP value of each bee pollen extract was calculated using the following equation:

$$\mathrm{FRAP}\;(µM\;\mathrm{Trolox}/g)=\;\frac{A_{s}-A_{b}}{S}\times D$$

where

$A_{s}$ = absorbance of the sample at 593 nm;

$A_{b}$ = absorbance of the blank (reagent without sample);

S = slope of the Trolox calibration curve (µM^−1^);

D = dilution factor, adjusted for sample concentration and extraction volume.

The calibration curve was constructed using Trolox (6-hydroxy-2,5,7,8-tetramethylchroman-2-carboxylic acid) as a standard antioxidant in concentrations ranging from 100 to 1000 µM. The results were expressed as µM Trolox equivalents per gram of bee pollen (µM TE/g).

### 2.8. Assessment of Mineral Composition

Approximately 0.2–0.3 g of each homogenized and dried bee pollen sample was accurately weighed and subjected to acid digestion following the procedure described by Caroli et al. [42], with minor modifications. Each sample was digested in a Teflon-coated closed vessel with 9 mL of concentrated nitric acid (HNO_3_, 65%) and 2 mL of hydrogen peroxide (H_2_O_2_, 30%) using a microwave-assisted digestion system (CEM MARSX Press, Matthews, NC, USA). The digestion continued until a clear, particle-free solution was obtained. After cooling, the digests were quantitatively transferred into 50 mL volumetric flasks and diluted to volume with ultrapure deionized water. Mineral quantification was performed using a microwave plasma–atomic emission spectrometer (MP–AES, Agilent 4210) for potassium (K), phosphorus (P), magnesium (Mg), manganese (Mn), calcium (Ca), sodium (Na), zinc (Zn), copper (Cu), and iron (Fe). Calibration curves were prepared from 1000 mg/L stock standard solutions of each element in 10% HNO_3_ and diluted with ultrapure water. All mineral concentrations were expressed as mg/kg of dry weight (DW). Total mineral content was calculated as the sum of all quantified elements. The analyses were performed in collaboration with the Center for Scientific and Technological Research Support (CACTI), University of Vigo.

### 2.9. Statistical Analysis

The Statistical analyses were carried out using STATGRAPHICS Centurion XVIII (Statgraphics Technologies, Inc., The Plains, VA, USA) and IBM SPSS V18 (IBM SPSS Statistics, Armonk, NY, USA). Shapiro–Wilk and Levene tests were used to verify data normality and homogeneity of variances, respectively. As some variables did not follow a normal distribution, non-parametric methods were applied where appropriate. Spearman’s rank correlation coefficients (ρ) were used to assess the relationships between variables and explore the associations among phenolic composition, antioxidant activity (RSA with DPPH assay, ABTS^+^•, and FRAP), protein and water content, mineral composition, and dominant pollen types. Only pollen types with relative frequencies greater than 40% in at least one sample were included in the statistical and multivariate analyses. Only correlations with *p* < 0.05 were considered statistically significant. A Principal Component Analysis (PCA) was performed on standardized data to explore patterns in the dataset and evaluate the relationships between biochemical, mineral, and botanical variables. According to the Kaiser criterion, components with eigenvalues ≥ 1 were retained. The loading plot was then used to visualize the contribution of each variable to the principal components, as well as to identify associations between pollen types and their biochemical or mineral characteristics. All statistical analyses were conducted at a 95% confidence level (*p* < 0.05).

## 3. Results

### 3.1. Botanical Origin of Bee Pollen Samples

The palynological analysis of bee pollen revealed the presence of 66 pollen types from 24 botanical families. The pollen profile revealed a clear foraging preference among honey bees for species belonging primarily to the Fabaceae, Brassicaceae, Cistaceae, and Apiaceae families. This reflects both the regional flora and the ecological plasticity of *Apis mellifera* when it comes to exploiting multiple floral sources. The Brassicaceae family was particularly well represented, with *Brassica napus* type (type onwards t.) reaching a maximum relative abundance of 78.1% (mean 13.2%) and being dominant in several samples. *Sinapis alba* also exhibited high representation (mean 4.8% and max 73.5%), confirming the attractiveness of Brassicaceae pollen. Other Brassicaceae, such as *Eruca sativa*, appeared less frequently, but reached up to 17.9% in one sample. Within the Cistaceae family, *Cistus* was one of the most prevalent and dominant pollen types (mean 10.9%, maximum 88.6%), which highlights the strong adaptation of bees to the flora of Mediterranean shrublands. The Fabaceae family also made a significant contribution to pollen composition, with *Hedysarum* leading the way (mean 13.5% and maximum 61.0%). *Onobrychis* (maximum 39.0%), *Trifolium repens* (maximum 9.0%), and *Lotus* (maximum 10.0%) represented additional, although less dominant, components. Within the Apiaceae family, the *Daucus carota* t. was the most prevalent (mean 5.9%, maximum 56%), followed by *Coriandrum sativum* (maximum 24%) and the *Ferula communis* t. (maximum 21.8%). The frequent occurrence of these pollen types is consistent with their wide distribution in open and disturbed Mediterranean habitats. Within Asteraceae, the *Anthemis* t. was predominant (maximum 31.7%), while the *Taraxacum* and *Inula* types reached 34.3% and 9.0%, respectively. The pollen of *Papaver* t. (Papaveraceae) appeared in a considerable number of samples (mean 2.6% and maximum 19.8%), confirming its regular, although secondary, contribution to the overall pollen profile. Several other pollen types, although not dominant, were consistently present and likely served as complementary or transitional foraging sources. These include *Olea europaea* (max. 25.2%), *Bryonia dioica* (max. 19.6%), *Myrtus communis* (max. 55.5%), *Quercus* (max. 29.1%), *Crataegus* (max. 48.0%), and *Acacia* (max. 45.4%). The diversity and distribution of pollen types showed that honey bees in the Mediterranean region of Algeria did not depend on a single main floral source. None of the bee pollen samples had more than 80% of one pollen type, which would classify them as monofloral. Instead, the results indicated that bees in this region collect pollen from a wide variety of plants. This diverse foraging behavior likely helps maintain the nutritional balance of bee colonies.

**Figure 2:**
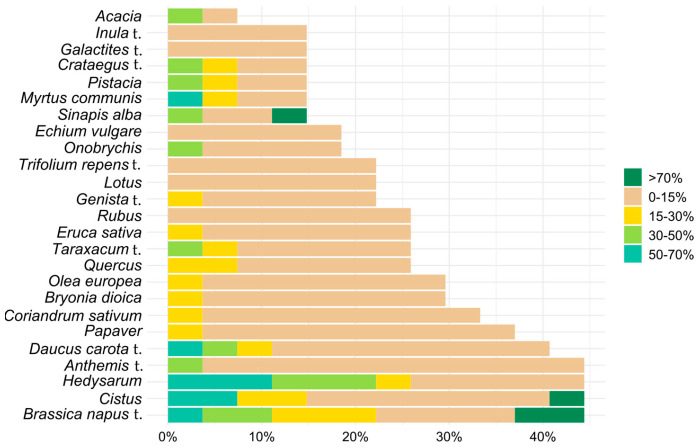
Frequency and relative abundance classes of the main pollen types identified in bee pollen samples.

In terms of frequency, 25 types of pollen were found in over 15% of bee pollen samples. Figure 2 shows the frequency with which the main pollen types occurred across all analyzed bee pollen samples, as well as their relative abundance classes, which are represented by color. The percentage on the *x*-axis indicates the frequency with which each pollen type was detected, while the color scale represents its abundance within individual samples. Of the pollen types identified, *B. napus* t. was the most frequently detected, appearing in over 40% of the analyzed samples. It was highly dominant in several cases, occurring at an abundance of 50–70% in 4% of samples and exceeding 70% in nearly 5%. *Cistus* and *Hedysarum* also appeared frequently, with high content in multiple samples, with *Cistus* reaching values above 70% in a few cases. *Anthemis* and *D. carota* showed moderate frequencies, occasionally appearing with contents between 15% and 50%. Other frequent pollen types, including *A. communis*, *Pistacia*, *Crataegus*, and *S. alba*, were found in fewer samples but sometimes reached high relative abundances.

### 3.2. Physisochemical Parameters: Water Content, Protein Content, and pH

The main physicochemical parameters of the bee pollen samples are summarized in Table 1. The water content averaged 24.4%, ranging from 12.5% to 32.7%. This indicates that differences in moisture content may be related to the pollen’s state of conservation or floral origin.

The protein content of the bee pollen samples studied showed high variability, with a coefficient of variation of 38% (mean value of 17.2 g/100 g dry weight (DW) and a standard deviation of 6.5). The highest protein content (over 28.0 g/100 g) was found in two samples with a high proportion of *Hedysarum* pollen (56.6% and 58.7%). In contrast, the lowest value (5.6 g/100 g DW) was found in the bee pollen sample with a high presence of *Taraxacum* t.

In terms of pH, the mean value was 4.5 ± 0.5, with a wide range of 3.8–6.0, indicating a coefficient of variation (CV) of 10.2%. The *Cistus* bee pollen sample had the lowest value compared to other bee pollen samples.

### 3.3. Total Phenolic Content, Total Flavonoid Content, and Antioxidant Activities

The results of functional properties extracted from the bee pollen samples are shown in Table 2. The total phenolic content (TPC) and total flavonoid content (TFC) of the 27 bee pollen samples showed substantial variability depending on botanical origin. The mean TPC was 1477.5 ± 325.5 mg GAE/100 g, with moderate variability (CV: 22.0%) and a range between 782.1 and 2167.4 mg GAE/100 g. The mean TPC varied markedly among the different types of bee pollen samples. The highest value (2167.4 mg GAE/100 g) was recorded in a bee pollen sample with *B. napus* t. pollen as the main pollen type (32.4%), followed by samples with a high content of *Acacia* (45.4%) (1888.3 mg GAE/100 g) and *M. communis* (55%) (2031.5 mg GAE/100 g). In contrast, the lowest content was found in bee pollen of *Hedysarum* (61%), with a value of 782.1 mg GAE/100 g.

The TFC also exhibited pronounced variability among samples, ranging from 78.1 to 766.8 mg QE/100 g, with a mean of 259.2 ± 127.5 mg QE/100 g (CV = 49.2%). The highest TFC was measured in bee pollen, dominated by *M. communis* (55.4%), reaching 766.8 mg QE/100 g, followed by samples rich in *Acacia* (45.4%) and *Daucus carota* t. (56.0%) with 445.6 and 324.2 mg QE/100 g, respectively. Other samples, such as those containing *Cistus* (27.0%) and *B. napus* t. (33.7%), also presented elevated TFC values (402.5 and 306.6 mg QE/100 g, respectively). In contrast, the lowest TFC was found in bee pollen, with 38.0% of the pollen from *Hedysarum* containing 78.1 mg QE/100 g. This indicates its limited contribution to the flavonoid content.

The antioxidant capacity of the bee pollen samples was evaluated using three standard assays: RSA, ABTS^+^•, and FRAP (Table 2). The mean values obtained for RSA, ABTS, and FRAP were 66.3 ± 18.9%, 52.7 ± 19.1%, and 142.4 ± 32.1 µmol Fe^2+^/g, respectively, with coefficients of variation above 25%, indicating pronounced heterogeneity among samples. Such differences are consistent with the variable floral origins of Algerian bee pollen, in which phenolic composition and antioxidant efficiency are directly influenced by the plant species visited by bees.

The radical scavenging activity evaluated by the DPPH assay (expressed as RSA) showed a mean inhibition of 66.1% ± 18.9, with a coefficient of variation of 28.5%, indicating moderate variability among the samples (Table 2). The values ranged from 27.2% to 86.0%, suggesting a wide range of free radical neutralization abilities across different pollen types. Among the samples analyzed, those with a high content of *B. napus* pollen type exhibited the highest and most consistent antioxidant activity, with a mean RSA inhibition of 84.9 ± 1.2%, and a coefficient of variation of only 1.4%, indicating very little intra-group variability. This suggests that *B. napus* t. contributes significantly to the antioxidant capacity of bee pollen. Samples rich in *S. alba* and in the mix of *B. napus* t./*Tamarix* also showed high antioxidant activity by RSA, with inhibition values of 86.0% and 85.5%, respectively, reinforcing the trend observed for the Brassicaceae family. In contrast, the samples dominated by *Hedysarum* presented a broader variability, with RSA values ranging from 27.2% to 81.4%, a high standard deviation (19.2%), suggesting strong compositional heterogeneity among the samples of this group. Samples with predominance of *Cistus* and *M. communis* showed moderate antioxidant activity, with mean RSA values of 74.2% and 70.4%, respectively. Lower antioxidant activity was found in samples rich in *D. carota* t. (average of 46.0%) and *Crataegus* t. (36.7%), indicating a lower radical scavenging ability associated with these pollen types.

The ABTS^+^• assay also revealed considerable variability, with a mean inhibition of 50.8% ± 20.4 and a high coefficient of variation (40.2%) (Table 2). The range of inhibition ranged from 22.8% to 93.7%, which reflects a broader heterogeneity in the electron-donating ability of the compounds present in the bee pollen extracts. Notably, ABTS^+^• showed the highest variability, suggesting that this method may be more sensitive to the diversity in chemical profiles of different pollens. The percentage of radical scavenging activity expressed as ABTS^+^• showed notable variation depending on the predominant pollen type found in each sample. Samples with *B. napus* t. as the dominant pollen showed the highest average ABTS inhibition (average of 74.7%), with values ranging from 41.9% to 93.7%, reflecting moderate variability (CV = 27.9%). The samples dominated by *Cistus* also demonstrated notable antioxidant activity (average of 59.4%, CV = 20.5%). *Hedysarum*-rich samples exhibited moderate ABTS^+^• inhibition (average of 46.3%), but with high variability (CV = 39.3%), indicating differences in sample composition or secondary pollen contributions within this group. Some individual samples stood out significantly. The sample with *S. montanum* as the dominant pollen type showed the highest ABTS^+^• inhibition (89.3%), despite being represented by a single sample. Likewise, *S. alba* (68.5%) and *Taraxacum* t. (66.2%) showed high antioxidant capacities, supporting the role of specific wild taxa in contributing to functional properties. On the other hand, samples dominated by *Onobrychis*, *Pistacia*, and *D. carota* t. showed lower inhibition values (mean = 28.8%, 28.6%, and 29.0%, respectively), with low intra-group variability where applicable. These results indicate a more limited antioxidant potential in these pollen types. The samples with *M. communis* showed low to moderate ABTS^+^• activity (average of 41.6%, CV = 10.9%), contrasting with its relatively higher RSA values observed in the previous analysis, suggesting that different antioxidant mechanisms may be favored depending on the radical species assessed.

The FRAP values averaged at 13.8 ± 3.9 µmol Trolox/g, with a CV of 28.1% (Table 2). Results ranged from 8.5 to 21.2 µmol Trolox/g, indicating a moderate but consistent ferric ion reducing capacity in most samples. Compared to ABTS^+^•, this assay suggests more consistent activity across the samples. However, the antioxidant capacity determined by the FRAP assay showed substantial differences depending on the predominant pollen type in the samples analyzed. Samples dominated by *B. napus* t. had the highest mean values (19.1 μmol Trolox/g) and low variability (CV = 8.0%), which is consistent with their high levels also reported in the other essays. This supports the idea that Brassicaceae pollen provides a significant content of compounds with reducing capacity, such as polyphenols and flavonoids. In contrast, pollen types such as *Hedysarum* (10.6 μmol Trolox/g) and *D. carota* t. (10.2 μmol Trolox/g) exhibited lower reducing capacities, along with *Crataegus* t. (10.6 μmol Trolox/g) and *Taraxacum* t. (11.2 μmol Trolox/g). Also noteworthy is the behavior of *M. communis*, whose samples presented highly divergent values (8.5 and 18.5 μmol Trolox/g), with a high coefficient of variation (CV = 52.4%).

### 3.4. Mineral Composition

The total mineral content of the analyzed pollen samples varied significantly depending on the predominant pollen type. Values ranged from 7598.1 to 16,414.1 mg/kg, with an overall mean of 12,210.9 mg/kg and a standard deviation of 2926.0 mg/kg (Table 3). The coefficient of variation was 24.0%, indicating moderate variability between samples. Although the bee pollen samples were not strictly monofloral, the predominant representation of certain pollen types showed differences in mineral content attributed to their botanical origin. The bee pollen sample with the highest total mineral value (15,696.2 mg/kg) was the one with the highest *Acacia* content. Samples dominated by *Hedysarum* (13,727.5 mg/kg) and *M. communis* (13,930.9 mg/kg) also exhibited high mineral content. The sample associated with *Cistus* presented the lowest total mineral value (9224.0 mg/kg) among those analyzed.

The most abundant mineral was K, with a mean content of 5289.2 mg/kg and low variability (CV = 13.5%), suggesting a relatively uniform distribution (Table 3). P and Mg had mean content values of 3732.7 mg/kg and 875.1 mg/kg, respectively, with moderate variability (CV of 31.2% and 35.7%, respectively). Ca exhibited greater dispersion (CV = 55.0%), indicating significant differences between samples. Fe and Cu exhibited high coefficients of variation, with values higher than 90%, indicating significant heterogeneity in their content according to pollen composition. Na and Zn also exhibited significant variability, with minimum values that were substantially lower than the maximum values recorded. These differences may be due to the botanical origin of the pollen and the edaphic and environmental conditions in which the producing plants developed.

At the botanical level, the highest total mineral content was recorded in samples dominated by *Acacia* (45.4%), reaching 15,696.2 mg/kg. This pollen type was particularly rich in Ca (3576.5 mg/kg) and K (5978.2 mg/kg), as well as showing elevated P (4289.4 mg/kg) and Mg (1230.2 mg/kg) levels, suggesting that *Acacia* contributes substantially to the macronutrient content of bee pollen. Similarly high concentrations were found in pollen dominated by *M. communis* (36.4%) and *Hedysarum* (48.7%), with total mineral values of 13,710.4 mg/kg and 13,727.6 mg/kg, respectively. *M. communis* samples were rich in K (6167.2 mg/kg) and P (4379.1 mg/kg), whereas *Hedysarum* pollen stood out for its high P (4914.6 mg/kg) and Cu (30.9 mg/kg) contents, reflecting a micronutrient-accumulating ability of certain Fabaceae. Intermediate mineral levels were observed in pollen samples dominated by *B. napus* t. (total = 11,664.3 mg/kg) and *P. lentiscus* (13,975.7 mg/kg). *B. napus* pollen exhibited relatively balanced concentrations of P (3461.7 mg/kg), K (5032.4 mg/kg), and Mg (951.1 mg/kg), while *P. lentiscus* pollen was characterized by very high Ca (3066.4 mg/kg) and Fe (813.4 mg/kg) levels, the latter representing the maximum iron concentration found among all samples. Pollen samples rich in *S. montanum* also presented elevated mineral content (13,009.8 mg/kg), especially Ca (2427.6 mg/kg) and Fe (377.4 mg/kg). In contrast, *Taraxacum* t. pollen showed the lowest overall mineral content (7598.1 mg/kg) and the minimum levels of P (2043.4 mg/kg), Mg (398.0 mg/kg), and Fe (40.1 mg/kg). Similarly, a sample dominated by *Cistus* (58.2%) presented a low total mineral value with 9224.0 mg/kg, characterized by lower concentrations of P (2666.7 mg/kg) and Mg (605.9 mg/kg). Zinc was detected in a subset of bee pollen samples, with concentrations ranging from <26 to 58.7 mg/kg. The highest Zn levels were found in samples dominated by *Hedysarum* (58.7 mg/kg), followed by *Acacia* (46.3 mg/kg) and *B. napus* t. (45.6 mg/kg). Moderate levels were observed in *Cistus* (29.8 mg/kg) and *M. communis* (29.6 mg/kg). In several samples, zinc values were below the detection limit, indicating substantial variability likely related to both botanical and environmental factors. These results indicate that Fabaceae (*Hedysarum* and *Acacia*) and Myrtaceae (*M. communis*) pollen types are among the most mineral-rich sources. These findings confirm that the mineral profile of Algerian bee pollen is largely determined by its botanical composition. The association between high mineral content and taxa such as *Acacia*, *Hedysarum*, and *M. communis* reinforces their potential nutritional relevance in multifloral bee pollen from the Mediterranean region.

### 3.5. Relationships Between Botanical Origin, Phenolic Content, Flavonoid Content, Antioxidant Properties, and Minerals

**Figure 3:**
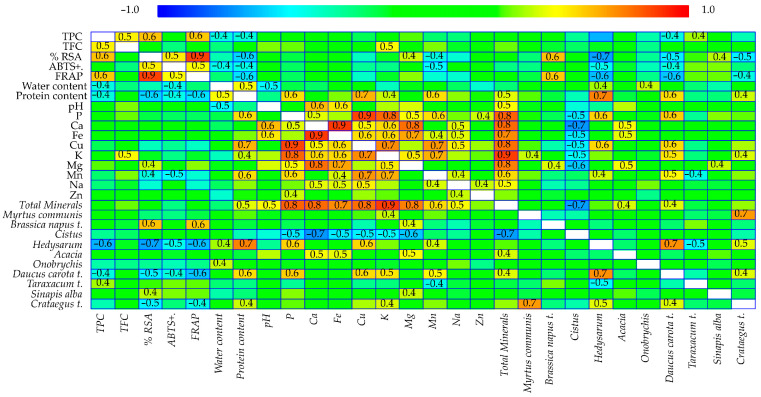
Spearman’s ordinal correlation between total mineral and protein content and antioxidant activity in bee pollen samples. The number shows a significant correlation at the 5% level.

Spearman’s rank correlation analysis was performed to explore the relationships among 28 variables, including antioxidant properties, physicochemical parameters, mineral composition, and the botanical origin of the pollen samples (Figure 3). The analysis revealed statistically significant correlations (*p* < 0.05), indicating meaningful relationships between both functional properties and pollen composition.

Strong positive correlations among TPC, TFC, radical scavenging activity (RSA%), and Ferric Reducing Antioxidant Power (FRAP) were found. This suggests that phenolic compounds play a central role in the antioxidant potential of the pollen samples. Additionally, TPC was positively correlated with water and protein content, as well as with the relative abundance of *D. carota*, *Hedysarum*, and *Taraxacum* t., indicating a botanical influence on the phytochemical profile. RSA showed significant correlations with ABTS inhibition, FRAP, and protein content, reinforcing the consistency among different antioxidant assays. Additionally, this variable exhibited positive correlations with *B. napus* and *S. alba* pollen types. A positive association was also found between ABTS inhibition and FRAP. Furthermore, FRAP values were correlated with the abundance of *B. napus* t. pollen, underscoring the fact that phytochemical content and botanical origin both influence redox activity. Beyond functional variables, water content was positively related to protein content and the pollen of *Hedysarum* and *Onobrychis*. There was a positive correlation between protein content and the abundance of *Crataegus*, *D. carota*, and *Hedysarum*.

The influence of botanical characteristics and bioactive components on the mineral composition of pollen collected by bees was also analyzed. Total mineral content showed significant positive correlations with protein content, pH, and the presence of *Acacia* and *D. carota* t. These relationships reinforce the integral role of floral origin in shaping biochemical traits and determining the nutritional value of pollen through its mineral composition. P exhibited strong positive correlations with protein content. It also showed significant associations with the presence of *D. carota* t. and *Hedysarum*. Therefore, these botanical sources notably contribute to the P profile of bee pollen. Ca was significantly correlated with pH. Furthermore, Ca exhibited positive associations with *Acacia* pollen types. Iron (Fe) demonstrated significant correlations with pH and *Acacia* pollen types, suggesting that these taxa may influence the bioavailability or accumulation of iron in pollen. As Fe plays an important role in redox reactions, its botanical source may be linked to antioxidant function. Cu was positively associated with protein content. Additionally, Cu showed strong correlations with *D. carota* and *Hedysarum*, suggesting that these plants contribute to the micronutrient richness of pollen. These associations are particularly interesting when considering the role of Cu in antioxidant enzymes. K was positively correlated with both TFC and protein content, reflecting its central role in plant metabolism and secondary compound synthesis. K also showed significant associations with several botanical pollen types, including *Crataegus* t., *D. carota* t., and *M. communis*, indicating that these are major sources of potassium-rich pollen. These relationships emphasize the link between botanical origin and functional bioactivity in pollen. Mg showed significant positive correlations with RSA, *Acacia*, *B. napus* t., and *S. alba*. Mn was positively associated with protein content, *D. carota* t., and *Hedysarum*.

**Figure 4:**
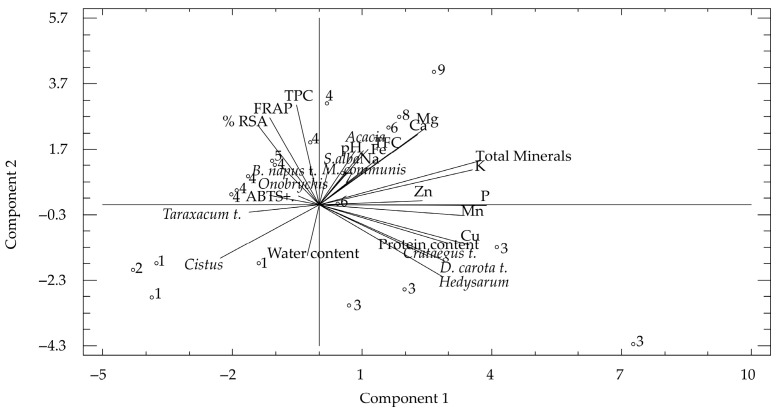
A biplot of the first two principal components illustrates the relationships among biochemical, mineral, and botanical variables in bee pollen samples. Samples were labeled (1–9) according to their dominant pollen type: (1) *Cistus*; (2) *Taraxacum* t.; (3) *Hedysarum*; (4) *B. napus* t.; (5) *S. alba*; (6) *Pistacia*; (7) *S. montanum*; (8) *M. communis*; and (9) *Acacia*.

Principal component analysis was conducted on 28 variables: TPC, TFC, antioxidant activities, water content, protein content, individual minerals, and botanical variables of bee pollen (Figure 4). Seven components with eigenvalues greater than 1 were extracted, explaining 88.2% of the total variance in the dataset. The first two components accounted for nearly 50% of the variance (28.2% and 19.2%, respectively), providing a reliable summary of the main patterns in the data. The biplot of the first two components (Figure 4) highlights the distinct clustering of biochemical and mineral variables. Component 1 was mainly influenced by mineral variables (P, K, Cu, Mn, and total minerals), along with protein content and specific botanical taxa such as *D. carota* t. and *Hedysarum*. In contrast, Component 2 was driven by antioxidant-related traits, with high loadings for TPC, RSA, FRAP, and TFC. Botanical taxa also showed associations with specific compositional patterns. For instance, *Acacia*, *B. napus* t., and *M. communis* loaded positively on Component 2, aligning with antioxidant variables, while *Crataegus* t., *D. carota* t., and *Hedysarum* clustered closer to Component 1, together with mineral and protein variables. This reflects the botanical influence on both nutritional and functional profiles of bee pollen. Although the Kaiser–Meyer–Olkin (KMO) test indicated low sampling adequacy, PCA still provided a useful exploratory framework to identify underlying relationships among biochemical, mineral, and botanical traits. Additionally, while the PCA did not strictly classify samples by floral origin, the distribution pattern suggests that moderate contributions from certain pollen types can influence biochemical and mineral composition. This indicates that the botanical origin of pollen imparts distinctive nutritional and antioxidant characteristics, enabling pollen groups to be differentiated to some extent based on their dominant floral source.

## 4. Discussion

Bee pollen has long been recognized in alternative medicine and dietary practices for its nutritional value and positive health effects [28,29]. In addition to its nutritional richness, it possesses a wide array of biological properties, including antibacterial activity [16], as well as anti-mutagenic and anti-inflammatory properties [11]. Of particular interest is its high antioxidant activity, which is mainly attributed to the presence of phenolic and flavonoid compounds [2]. However, bee pollen is far from uniform in characterization, as it is strongly influenced by geographical and botanical origins, climatic conditions, soil type, beekeeping practices, and storage methods [43].

This study comprehensively characterized bee pollen samples from the Mediterranean region of Algeria through palynological analysis, quantification of phenolic and flavonoid content, assessment of antioxidant activity, and analysis of mineral composition. Palynological identification confirmed that the botanical origin exerts a decisive influence on pollen properties. Families such as Apiaceae, Asteraceae, Brassicaceae, Cistaceae, Fabaceae, and Papaveraceae were predominant, in line with the richness of Mediterranean flora [43]. The most frequent dominant pollen types were *Cistus* (Cistaceae), *B. napus* (Brassicaceae), *Hedysarum* (Fabaceae), *Crataegus* (Rosaceae), *D. carota* (Apiaceae), *Silybum marianum* (Asteraceae), *M. communis* (Myrtaceae), and *Acacia* (Mimosaceae). These findings are consistent with previous studies conducted in Algeria [33] and Morocco [8,44]. These findings confirm the close link between floral composition and the physicochemical and biological traits of bee pollen.

Phenolic and flavonoid compounds are key bioactive constituents of bee pollen with well-established health benefits, including reducing oxidative stress and inhibiting macromolecular oxidation [16]. The levels of total phenols detected in this study were generally higher than those previously reported for Algerian bee pollen samples [13] but were similar for Spanish [12] and Brazilian samples [8,45]. Compared with Moroccan bee pollen [8,46] and commercial and Romanian samples [11,19], the present results confirmed a richer profile in terms of TPC and TFC, underscoring the bioactive potential of Algerian bee pollen.

The antioxidant and mineral profiles of bee pollen collected from the Mediterranean region of Algeria demonstrate the significant impact of botanical origin on its biochemical and nutritional properties. Due to the complex and multifactorial nature of antioxidant mechanisms [7,47], the assessment of antioxidant activity through complementary assays, such as RSA with a DPPH assay, ABTS, and FRAP, was essential. The high radical scavenging and reducing capacities observed confirm that phenolic compounds and flavonoids are the main contributors to the antioxidant potential of bee pollen. This is consistent with previous findings relating to European and Mediterranean samples [12].

The positive correlations observed between TPC and antioxidant assays (RSA and FRAP) suggest that the redox potential of bee pollen is largely dependent on its phenolic composition. However, discrepancies among different antioxidant tests suggest that these compounds act through diverse mechanisms, with their reactivity varying depending on the polarity and structure of individual molecules [2,14]. This confirms the importance of applying a multi-methodological approach to accurately assessing antioxidant potential.

Botanical composition was found to be a key determinant of functional activity. Pollen types such as *B. napus*, *M. communis*, *Acacia*, and *S. alba* exhibited stronger antioxidant profiles, likely due to their high polyphenol and flavonoid content. In contrast, *Hedysarum* and *D. carota* contributed mainly to protein content rather than antioxidant capacity. These findings support the idea that the diversity of flowers directly influences the nutritional and bioactive composition of bee pollen [12,20,22].

The mineral composition further confirmed the strong link between botanical origin and nutritional value. Macronutrients such as potassium, phosphorus, calcium, and magnesium predominated, which is consistent with the findings of previous studies in Mediterranean and temperate regions [10,24]. Pollen from Fabaceae and Myrtaceae species, particularly *Acacia*, *Hedysarum*, and *M. communis*, had a high mineral content. This reflects the plants’ ability to accumulate essential nutrients through symbiotic and physiological processes [9,27]. This highlights the plants’ capacity to accumulate essential nutrients via various biological mechanisms. This mineral richness is due to symbiotic relationships and physiological adaptations, making the pollen valuable for ecological and nutritional purposes. For instance, *Acacia* pollen has been demonstrated to contain substantial quantities of potassium, calcium, phosphorus, and magnesium, which are vital for human health and nutrition [48,49]. It can also be used as a dietary supplement thanks to its high iron content, helping to fulfill daily nutritional requirements [48]. By contrast, pollen from *Cistus* and *Taraxacum* tended to have lower mineral levels, which may reflect an adaptation to less fertile or sandy soils, as has been observed in other Mediterranean ecosystems. This adaptation is evident in their capacity to flourish in environments where nutrient availability is restricted, thereby influencing their mineralization processes. In particular, *Cistus* has been shown to affect soil nutrient dynamics in ways that may contribute to lower mineralization levels, such as reducing nitrogen and magnesium availability under its canopy [50]. This behavior is consistent with the nutrient-poor conditions of Mediterranean ecosystems, where plants have evolved specific strategies to cope with limited resources.

Correlation and multivariate analyses provided deeper insights into these interrelations. The strong associations observed between phenolic compounds, antioxidant responses, and mineral content demonstrate that the bioactive potential of bee pollen is determined by both its chemical composition and its botanical structure. Elements such as potassium and copper were found to be related to phenolic and protein content, thereby reinforcing their role in secondary metabolism and antioxidant enzyme systems [9,23]. Integrating mineral and antioxidant data through principal component analysis also highlighted the complementary influence of floral and edaphic factors on the nutritional complexity of bee pollen.

Overall, these findings establish Algerian bee pollen as a product of significant nutritional and functional value. Its compositional diversity, driven by its multifloral botanical origin, reflects the environmental and ecological variability of Mediterranean landscapes. These results are consistent with international literature, supporting the potential of bee pollen as a natural source of bioactive compounds and essential minerals [7,10,19]. Further research integrating analyses of amino acid, vitamin, and mineral bioavailability would improve our understanding of its role as a functional food and as an indicator of the ecological quality of Mediterranean ecosystems.

## 5. Conclusions

Bee pollen samples collected from the Mediterranean region of Algeria were characterized using palynological, biochemical, mineral, and antioxidant analyses. The results showed that botanical origin strongly influences the biochemical and mineral composition of bee pollen, as well as its antioxidant potential. The most promising sources were identified as pollen types such as *B. napus* t., *M. communis*, and *Acacia*, which exhibited the highest TPC, TFC, and antioxidant activity (RSA, ABTS, and FRAP), as well as elevated levels of key minerals such as K, P, Ca, and Mg. In contrast, pollen dominated by *Hedysarum* and *D. carota* showed comparatively lower phenolic and antioxidant values, but higher protein and total mineral content, suggesting a complementary nutritional value. The mineral analysis revealed substantial variability linked to floral origin. *Acacia*, *M. communis*, and *Hedysarum* pollen had the highest total mineral content and were particularly rich in K, P, and Ca, while *Cistus* and *Taraxacum* t. had lower overall mineral levels. This compositional diversity reflects the botanical affinity and environmental adaptation of the floral sources, thereby reinforcing the multifloral nutritional value of Algerian bee pollen.

These findings confirm that bee pollen from Algeria’s Mediterranean region constitutes a natural product that is both nutritionally valuable and functionally active, combining antioxidant potency with essential minerals and proteins. The results highlight the potential of specific taxa as indicators of nutritional quality and suggest that integrated biochemical-mineral profiling can be used to reliably assess the authenticity and functional potential of bee pollen. Further studies incorporating analyses of seasonality, environmental factors, and bioavailability are recommended to improve our understanding of the interactions between the botanical origin, mineral composition, and biological activity of bee pollen, and to promote its use as a functional food ingredient and natural dietary supplement.

## Figures and Tables

**Table 1:** Water and protein content of bee pollen samples. SD: standard deviation; CV: coefficient of variation.

	Average	SD	CV (%)	Minimum	Maximum
Water content (%)	24.4	5.5	22.4	12.5	32.7
Protein content (g/100 g DW)	17.2	6.5	38.0	5.6	28.9
pH	4.5	0.5	10.2	3.8	6.0

**Table 2:** Functional properties of bee pollen samples. TPC: total phenol content; TFC: total flavonoid content; RSA: radical scavenging activity; FRAP: Ferric Reducing Antioxidant Power; SD: standard deviation; CV: coefficient of variation.

	Average	SD	CV (%)	Minimum	Maximum
TPC (mg GAE/100 g)	1477.5	325.5	22.0	782.1	2167.4
TFC (mg QE/100 g)	259.2	127.5	49.2	78.1	766.8
RSA (%)	66.1	18.9	28.5	27.2	86.0
ABTS^+^• (%)	50.8	20.4	40.2	22.8	93.7
FRAP (μmol Trolox/g)	13.8	3.9	28.1	8.5	21.2

**Table 3:** Mineral composition of bee pollen samples.

Minerals (mg/kg)	Average	SD	CV (%)	Minimum	Maximum
K	5289.2	713.1	13.5	4055.6	6411.2
P	3732.7	1064.9	31.2	2043.4	6544.3
Ca	1921.1	1056.5	55.0	711.4	3959.0
Mg	875.1	312.1	35.7	398.0	875.1
Fe	227.2	220.6	97.1	40.1	1152.6
Na	112.6	53.0	47.0	29.3	313.9
Zn	39.3	18.3	46.5	<26	58.7
Mn	25.3	10.1	40.0	10.5	38.8
Cu	14.5	13.0	91.4	5.4	42.1
Total	12,210.9	2926.0	24.0	7598.1	16,414.1

## Data Availability

The original contributions presented in this study are included in the article. Further inquiries can be directed to the corresponding author.
